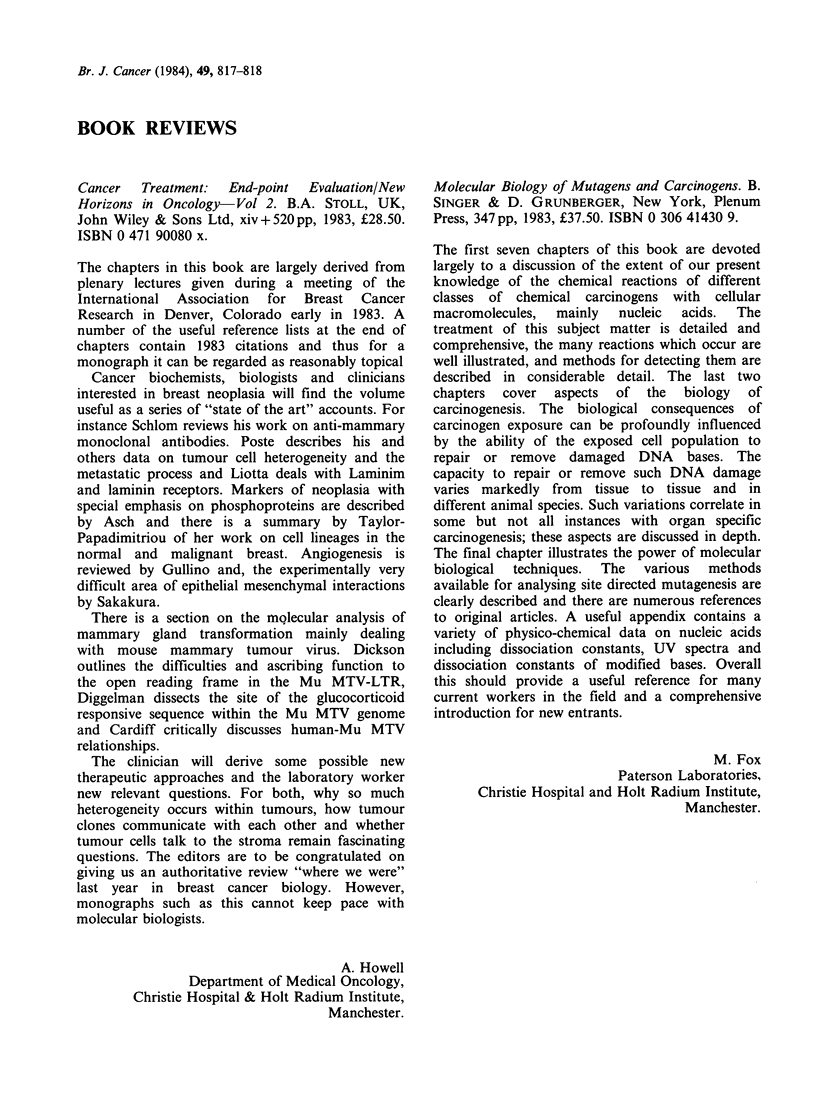# Molecular Biology of Mutagens and Carcinogens

**Published:** 1984-06

**Authors:** M. Fox


Manchester.

*Molecular Biology of Mutagens and Carcinogens.* B. Singer & D. Grunberger, New York, Plenum Press, 347 pp, 1983, £37.50. ISBN 0 306 41430 9.

The first seven chapters of this book are devoted largely to a discussion of the extent of our present knowledge of the chemical reactions of different classes of chemical carcinogens with cellular macromolecules, mainly nucleic acids. The treatment of this subject matter is detailed and comprehensive, the many reactions which occur are well illustrated, and methods for detecting them are described in considerable detail. The last two chapters cover aspects of the biology of carcinogenesis. The biological consequences of carcinogen exposure can be profoundly influenced by the ability of the exposed cell population to repair or remove damaged DNA bases. The capacity to repair or remove such DNA damage varies markedly from tissue to tissue and in different animal species. Such variations correlate in some but not all instances with organ specific carcinogenesis; these aspects are discussed in depth. The final chapter illustrates the power of molecular biological techniques. The various methods available for analysing site directed mutagenesis are clearly described and there are numerous references to original articles. A useful appendix contains a variety of physico-chemical data on nucleic acids including dissociation constants, UV spectra and dissociation constants of modified bases. Overall this should provide a useful reference for many current workers in the field and a comprehensive introduction for new entrants.

M. Fox
Paterson Laboratories,
Christie Hospital and Holt Radium Institute,
Manchester.